# The Stent That Went Too Far: Ascending Colon Perforation Following Gastric Stent Migration

**DOI:** 10.7759/cureus.111948

**Published:** 2026-07-02

**Authors:** Hassan Zaheer, Sayed Moosa Kazim, Hassan Mohsin, Muhammad Nouman Rafique, Shahid Khattak, Aamir Ali Syed

**Affiliations:** 1 Surgical Oncology, Shaukat Khanum Memorial Cancer Hospital and Research Centre, Lahore, PAK

**Keywords:** colonic perforation, flot chemotherapy, gastric outlet obstruction, self-expanding metal stent, stent migration

## Abstract

A 32-year-old male patient presented with symptoms of gastric outlet obstruction secondary to distal gastric adenocarcinoma. A fully covered self-expanding metal stent (SEMS) measuring 20 mm × 130 mm was placed to relieve obstruction and restore oral intake, with the aim of optimizing the patient for planned perioperative chemotherapy. The patient subsequently received neoadjuvant FLOT (fluorouracil, leucovorin, oxaliplatin, and docetaxel) chemotherapy as per recommendations of the multidisciplinary team and completed three cycles uneventfully. Before scheduled restaging imaging and completion of the fourth cycle, he developed sudden-onset abdominal pain with signs of generalized peritonitis. Imaging revealed pneumoperitoneum, and an emergency exploratory laparotomy was performed. Intraoperatively, a perforation was identified in the ascending colon near the hepatic flexure. A right hemicolectomy was carried out. Histopathological examination demonstrated a foreign body consistent with a migrated stent embedded at the perforation site, confirming complete transintestinal migration of the gastric stent as the cause of colonic perforation. This case highlights a rare but serious complication of fully covered gastric SEMS, particularly in patients receiving neoadjuvant chemotherapy, and emphasizes the need for clinical vigilance even in the absence of interim symptoms.

## Introduction

Gastric outlet obstruction is a frequent complication of advanced distal gastric cancer, resulting in persistent vomiting, malnutrition, and inability to tolerate oral intake. Endoscopic placement of self-expanding metal stents (SEMS) has become a widely accepted, minimally invasive option for rapid palliation and restoration of oral intake. It is also increasingly used as a bridge to systemic therapy and surgery, particularly in patients planned for perioperative chemotherapy [1].

Compared with surgical gastrojejunostomy, SEMS placement allows faster recovery, shorter hospital stay, and earlier initiation of oncological treatment [2,3]. In patients with resectable gastric cancer, perioperative chemotherapy, most commonly the FLOT regimen (fluorouracil, leucovorin, oxaliplatin, and docetaxel), followed by surgical resection has become the standard treatment approach in many centres, improving survival outcomes in selected patients [4].

In addition to its clinical benefits, SEMS placement is associated with procedure-related complications. Among these, stent migration remains one of the most important and clinically relevant adverse events. Migration is significantly more frequent with fully covered SEMS compared with uncovered or partially covered designs due to the absence of tumor ingrowth and reduced anchoring to the bowel wall. Reported migration rates range from approximately 8% to 25%, depending on stent type, patient selection, and follow-up duration [5,6].

Beyond device-related factors, several physiological and treatment-related mechanisms may contribute to stent migration. Restoration of luminal patency following decompression, combined with partial tumor regression after chemotherapy, may alter intraluminal forces and reduce fixation of the stent within the malignant stricture. Fully covered stents, while advantageous in preventing tumor ingrowth and facilitating potential removal, therefore carry an inherent trade-off of increased mobility within the gastrointestinal tract [7,8].

Although most migrated stents pass through the gastrointestinal tract without clinical consequence, clinically significant complications are increasingly recognized. These include intestinal obstruction, fistula formation, and, in rare cases, bowel perforation. Reported perforations most commonly involve the sigmoid colon and are predominantly associated with biliary stents rather than gastric SEMS. Gastrointestinal perforation secondary to migrated stents remains an uncommon but serious event and is best described in isolated case series and multicentre analyses [9,10].

To our knowledge, only a handful of such cases have been documented, and none have described perforation of the ascending colon specifically.

Consequently, colonic perforation following complete transintestinal migration of a gastric SEMS represents an exceptionally rare clinical scenario. The limited number of reported cases highlights the need for continued awareness of this complication, particularly in patients receiving neoadjuvant chemotherapy after stent placement.

## Case presentation

A 32-year-old male patient presented with a three-month history of progressive vomiting, epigastric pain, and weight loss. Upper gastrointestinal endoscopy revealed a stenosing distal gastric tumor involving the antrum and incisura. The scope could not be negotiated beyond the lesion. Biopsies confirmed moderately differentiated adenocarcinoma.

In patients with distal gastric malignancy causing obstructive symptoms, SEMS placement is indicated when the endoscope cannot traverse the tumor or passage is significantly difficult. The objective is to restore oral intake, improve nutritional status, and optimize the patient for systemic therapy.

A fully covered SEMS (20 mm × 130 mm) was placed across the obstructing lesion. Following multidisciplinary discussion, the patient was planned for perioperative chemotherapy using the FLOT regimen, consisting of four preoperative cycles followed by surgical resection and four postoperative cycles. Staging PET-CT was performed before initiation of chemotherapy and revealed a hypermetabolic circumferential gastric tumor in the antral region with a standardized uptake value (SUV) of 5.4, with the metallic stent in situ. A faintly avid 0.4 cm peritumoral node was noted anterior to the distal stomach/pylorus. No distant metastatic disease was identified. The disease was staged as T3N1M0 (Figure 1).

**Figure 1:**
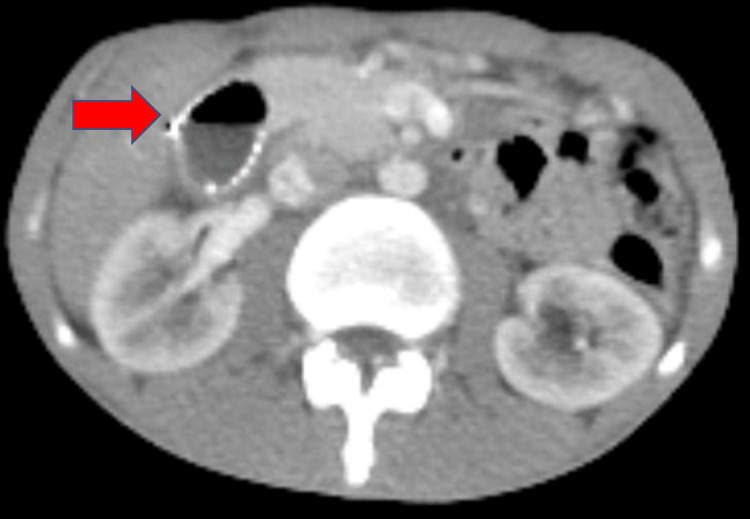
CT scan demonstrating the placement of a fully covered self-expanding metal stent (red arrow) across a malignant distal gastric stricture.

The clinical timeline from stent placement to the final follow-up is summarized in Table 1.

**Table 1:** Clinical timeline of key events FLOT: fluorouracil, leucovorin, oxaliplatin, and docetaxel

Event	Time Interval
Stent placement	Day 0
First cycle of FLOT chemotherapy	Day 22
Acute presentation with perforation	Day 52
Emergency right hemicolectomy	Day 52
Definitive distal gastrectomy	Day 156
Last follow-up (no recurrence)	~12 months post-gastrectomy

The patient completed three cycles of FLOT chemotherapy uneventfully. The fourth cycle and subsequent restaging PET-CT were scheduled but not yet performed at the time of acute presentation.

He then presented to the emergency department with sudden-onset abdominal pain and signs of generalized peritonitis. On presentation, the patient was hemodynamically stable with a heart rate of 82 beats per minute, blood pressure of 107/71 mmHg, temperature of 36.2°C, and respiratory rate of 20 breaths per minute. Laboratory investigations revealed a leukocytosis of 27.05 × 10³/µL (normal: 4.52-10.93), hemoglobin of 11.5 g/dL (normal: 13.2-16.7), and platelet count of 282 × 10³/µL (normal: 150-450). Serum electrolytes showed sodium 134 mmol/L (normal: 136-145), potassium 4.6 mmol/L (normal: 3.5-5.1), chloride 100 mmol/L (normal: 98-107), urea nitrogen 18.13 mg/dL (normal: 9-22), creatinine 0.45 mg/dL (normal: 0.90-1.30), estimated glomerular filtration rate (eGFR) 217.42 mL/min/1.73 m² (normal: >60), and bicarbonate 24.3 mmol/L (normal: 22-29).

An erect abdominal radiograph demonstrated pneumoperitoneum (Figure 2).

**Figure 2:**
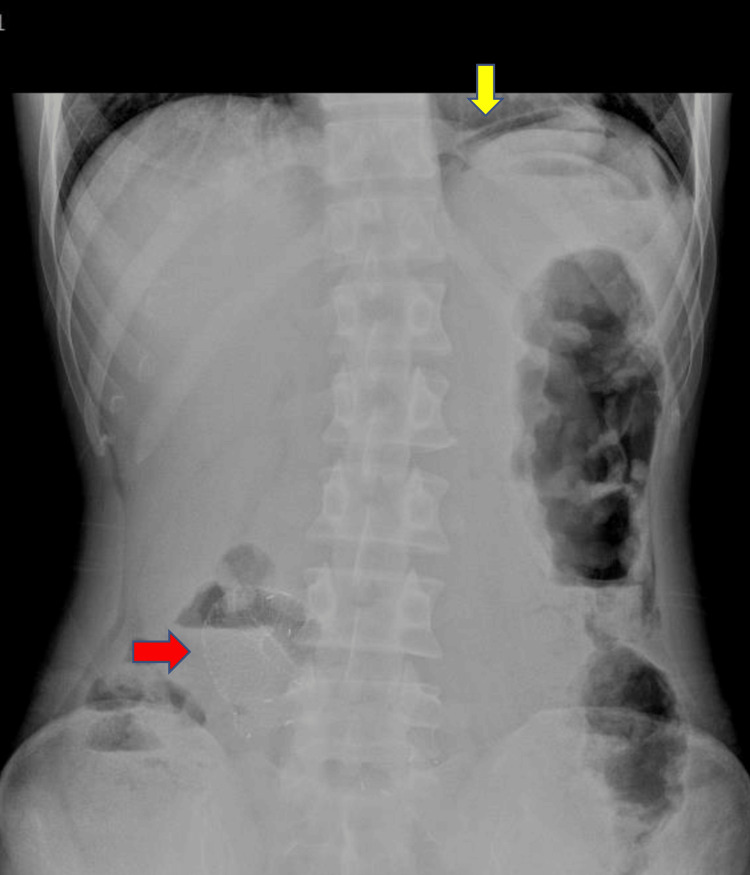
Erect abdominal radiograph showing free subdiaphragmatic air (yellow arrow) and a migrated self-expanding metal stent in the right lower abdomen (red arrow).

Emergency exploratory laparotomy was performed. Intraoperatively, no perforation was identified at the primary gastric tumor site. However, a perforation was found at the ascending colon near the hepatic flexure, with gross fecal contamination. Estimated blood loss was 50 mL, and operative duration was one hour and 43 minutes. No intraoperative complications occurred. A right hemicolectomy with peritoneal lavage was performed.

Histopathological examination of the right hemicolectomy specimen revealed transmural inflammation, mucosal ulceration with ulcer slough, and serosal exudative slough with necrotic debris and congested vessels, consistent with acute colonic perforation (Figures 3, 4). The gross histopathology report documented the presence of a foreign body consistent with the gastric stent embedded at the site of the transmural defect. One end of the stent was identified at the junction of the ascending and transverse colon, adherent to the perforation site, thereby confirming stent migration as the definitive cause of the perforation.

**Figure 3:**
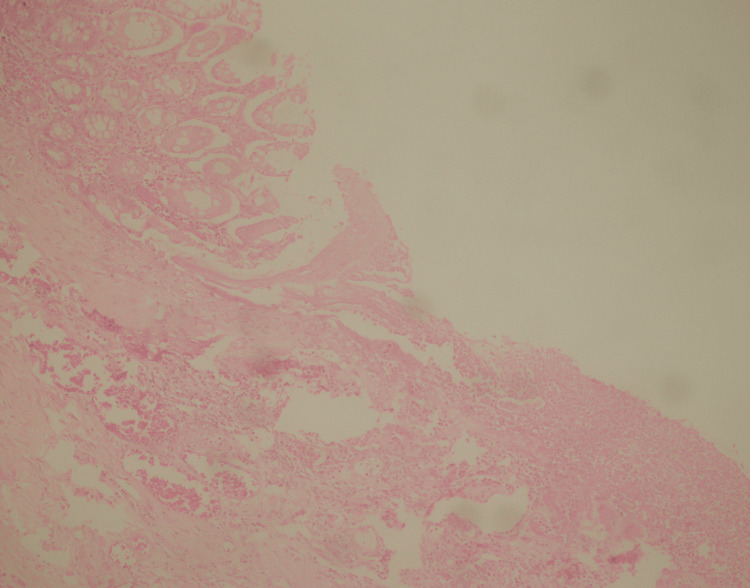
Histopathology slide showing colonic mucosa with ulceration, ulcer slough, and transmural inflammation extending through the bowel wall.

**Figure 4:**
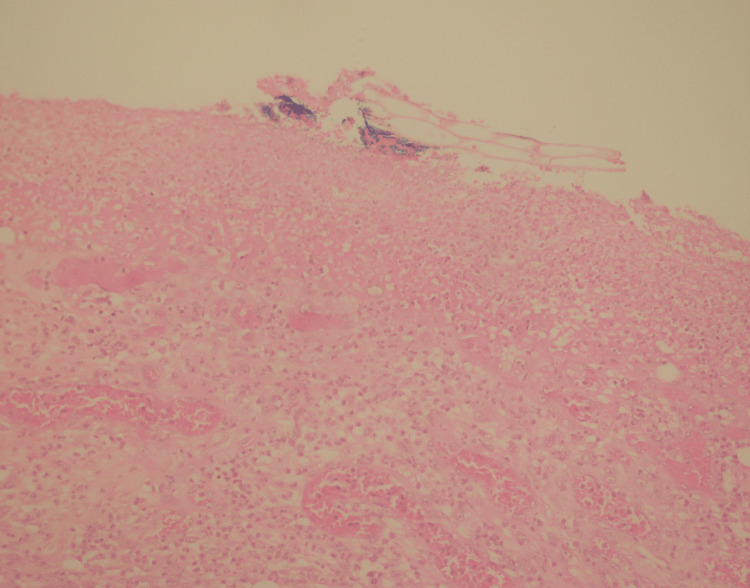
Histopathology slide showing serosa with exudative slough, necrotic debris, and congested vessels, consistent with acute perforation.

The patient had an uncomplicated postoperative course with a length of hospital stay of seven days. No postoperative complications such as wound infection, anastomotic leak, ileus, or need for ICU admission were observed.

Following recovery from the emergency right hemicolectomy, the patient received three additional cycles of FLOT chemotherapy (for a total of six neoadjuvant cycles), with a delay attributable to the emergency surgery. He subsequently underwent a laparoscopic-assisted distal gastrectomy with gastrojejunostomy and enteroenterostomy. Intraoperative findings included a tumor involving the antrum and pylorus, changes from previous right hemicolectomy, extensive omental and hepatic adhesions, moderate ascites, and no omentoperitoneal or visceral metastasis. Estimated blood loss was 100 mL, operative duration was five hours and 17 minutes, and an R0 resection was achieved. No intraoperative complications occurred. The postoperative course was uneventful, with a length of hospital stay of eight days.

The patient received two adjuvant cycles of FLOT chemotherapy after definitive surgery. At 12 months of follow-up, the most recent CT scan showed satisfactory postsurgical changes of gastrectomy without any residual or recurrent local disease or distant metastasis. The patient remains clinically well with no evidence of disease recurrence.

## Discussion

SEMS placement is an established modality for palliation of malignant gastric outlet obstruction and as a bridge to chemotherapy and surgery [1]. Its advantages include rapid symptom relief, improved nutritional status, and earlier initiation of systemic therapy compared to surgical bypass.

However, stent migration remains a clinically important limitation, particularly with fully covered SEMS. The absence of tumor ingrowth, while beneficial for stent removal, reduces fixation and increases the risk of distal migration [5,6].

In most cases, migrated stents pass through the gastrointestinal tract without causing harm. Rarely, however, they may act as rigid intraluminal foreign bodies leading to obstruction, erosion, or perforation [9]. The majority of reported perforations involve biliary stents, with the sigmoid colon being the most frequently affected site.

Comparison with previously reported cases

To contextualize our findings, we compared our case with previously published reports of colonic perforation secondary to stent migration. The available literature is summarized in Table 2.

**Table 2:** Summary of previously reported cases of colonic perforation secondary to stent migration GOO: gastric outlet obstruction; SEMS: self-expanding metal stent; FCSEMS: fully covered self-expanding metal stent; FLOT: fluorouracil, leucovorin, oxaliplatin, and docetaxel

Author (Year)	Stent Origin	Migrated Segment	Mechanism	Intervention
Kodia et al. (2022) [11]	Biliary	Ascending colon	Plastic biliary stent migration	Laparoendoscopic retrieval + repair
Gu et al. (2025) [12]	Biliary	Duodenum + ascending colon	Straight plastic stent transcolonic	Endoscopic retrieval + clipping
Beloy et al. (2024) [13]	Biliary	Sigmoid colon	Plastic biliary stent	Hartmann's procedure
Kaplan et al. (2014) [14]	Gastric (post-sleeve)	Small bowel	Stent for leak repair	Surgical resection
Fujikawa et al. (2024) [15]	Gastroduodenal	Small bowel	Fractured SEMS migration	Segmentectomy
Present case	Gastric (malignant GOO)	Ascending colon	FCSEMS + neoadjuvant FLOT	Emergency right hemicolectomy

As shown in Table 2, previously reported cases of colonic perforation from stent migration have predominantly involved biliary stents, with the sigmoid colon being the most frequently affected segment. Gastric stent migration, in contrast, has rarely been implicated in colonic perforation. Existing reports describe gastric stents migrating to the small bowel or sigmoid colon, but to our knowledge, no case of a gastric stent traversing the entire small intestine and perforating the ascending colon has been documented. Furthermore, our case is unique in that the stent was placed as a bridge to neoadjuvant chemotherapy in a patient with potentially resectable disease, rather than for palliative intent. The temporal association with FLOT chemotherapy and the absence of interim imaging add further clinical relevance, highlighting a potential blind spot in the surveillance of these patients.

In the present case, the stent likely dislodged following initiation of chemotherapy, when tumor regression reduced the anchoring effect of the malignant stricture. The patient had completed three cycles of FLOT, and the fourth cycle and planned restaging PET-CT were still pending. No interim imaging was performed, allowing silent migration to remain undetected.

The migrated stent traversed the entire gastrointestinal tract and impacted in the ascending colon, where it caused pressure necrosis and perforation. The length and rigidity of the 130 mm fully covered stent likely contributed to impaction at the ileocecal region and subsequent colonic injury.

This case highlights a potential risk in patients receiving neoadjuvant chemotherapy after SEMS placement, where tumor regression may possibly facilitate stent migration. Although no guidelines currently recommend routine interval imaging after SEMS placement in asymptomatic patients, this case raises the question of whether selective imaging during chemotherapy may be beneficial in high-risk cases.

Strengths and limitations

The key strength of this case is the histopathological confirmation of the stent at the perforation site, providing definitive evidence of causality rather than inferred association. A limitation of this case is the absence of interim imaging between stent placement and the acute presentation. While the temporal relationship between chemotherapy administration and perforation is suggestive, we cannot definitively prove that tumor regression was the sole mechanism of migration. Other factors, including peristaltic forces and the inherent mobility of fully covered stents, may have also contributed.

## Conclusions

Fully covered SEMS are effective for palliation of malignant gastric outlet obstruction and as a bridge to systemic therapy; however, they carry a risk of migration. This case demonstrates an extremely rare complication of complete transintestinal migration resulting in ascending colon perforation during neoadjuvant chemotherapy. Clinicians should remain vigilant for this possibility, particularly in patients receiving chemotherapy after stent placement.

Histopathological confirmation in this case strengthens the causal relationship and underscores the importance of awareness of this potentially life-threatening complication.
